# MiRNA-140-3p affects the proliferation and metastasis of osteosarcoma via the UBE2C/PI3K-Akt axis

**DOI:** 10.3389/fonc.2026.1742599

**Published:** 2026-07-29

**Authors:** Xu Yang, Yubo Xiang, Haoran Liu, Zhengchen Li, Zheng Luo, Jia Liu, Yongxin Bao, Yingxin Yang

**Affiliations:** 1 Department of Rehabilitation Medicine, General Hospital of Northern Theater Command, Shenyang, China; 2 Spinal Hospital of Enshi Tujia and Miao Autonomous Prefecture, Enshi, China; 3 Department of Orthopedic Surgery, The Forth Affiliated Hospital of China Medical University, Shenyang, China

**Keywords:** metastasis, miR-140-3p, osteosarcoma, PI3K/Akt pathway, UBE2C

## Abstract

The data of scRNA-seq in specific databases were analyzed by bioinformatics. The expression level of miR-140-3p in osteosarcoma cells was detected using qRT-PCR, and the correlation between the expression level of miR-140-3p and the survival of tumor patients was analyzed using a database. A series of experiments were conducted to explore the role of miR-140-3p in osteosarcoma cells. Database prediction of target genes, dual luciferase reporter gene experiments, and a series of *in vivo* and *in vitro* experiments were used to explore the related mechanisms of miR-140-3p’s influence on osteosarcoma proliferation and metastasis. MiR-140-3p was significantly lower expressed in osteosarcoma cells. Low expression of miR-140-3p is closely associated with poor survival of tumor patients. *In vitro* and *in vivo*, overexpression of miR-140-3p can significantly inhibit the proliferation and metastasis of osteosarcoma cells, hinder the cycle process of osteosarcoma cells, and promote the apoptosis of osteosarcoma cells. *In vivo*, overexpression of miR-140-3p can significantly inhibit subcutaneous tumor formation and lung metastasis of osteosarcoma cells in nude mice. Knockdown of miR-140-3p has the opposite effect *in vivo* and *in vitro*. Mechanistically, UBE2C is a direct target gene of miR-140-3p, and UBE2C is highly expressed in osteosarcoma cells and most cancer tissues, and is associated with poor survival of tumor patients. The interaction between miR-140-3p and UBE2C can affect the activation of the PI3K/Akt signaling pathway, which significantly changes the malignant biological characteristics of osteosarcoma cells *in vivo* and *in vitro*. The miR-140-3p/UBE2C/PI3K-Akt axis could be a promising target for treatment.

## 1 Introduction

Osteosarcoma is the most common primary malignant bone tumor, originating from bone marrow mesenchymal stem cells (1). Children and adolescents are the most common population of osteosarcoma, and the distal femur is the most common site of occurrence (1). These days, advances in adjuvant chemotherapy and surgical resection methods have led to significant improvements in the 5-year overall survival rate of osteosarcoma patients, increasing it from less than 20% to over 60% (2–4). However, due to the characteristics of invasive osteosarcoma, high lung metastasis rate and high recurrence rate, the therapeutic effect of osteosarcoma patients is still not up to expectations, especially in patients with recurrent and pulmonary metastatic osteosarcoma (5). Studies have found that the patients’ 5-year survival rate of those with osteosarcoma who have lung metastases at first examination is two times lower than those without lung metastases (4, 6, 7). Therefore, there is an urgent need to find early specific diagnostic biomarkers of osteosarcoma and effective therapeutic targets.

miRNA is a highly conserved endogenous non-coding single-stranded RNA that is transcribed from genes and is between 20–24 nucleotides (8). miRNA can affect the translation process of genes, regulate the synthesis of proteins, and participate in cell life activities such as proliferation, invasion, differentiation, apoptosis and autophagy, playing a key role in the formation, progression and metastasis of various tumors (9). In addition, the abnormal expression of miRNA is closely related to the clinical stage, pathological grade and overall survival rate of tumors (10). Among them, miR-140-3p is a tumor inhibitor in liver cancer, and the down-regulation of miR-140-3p enhances the malignant biological behaviors of liver cancer cells, such as proliferation, migration and invasion (11). Overexpression of miR-140-3p can also significantly inhibit the migration, invasion and epithelial mesenchymal transformation of gastric cancer cells (12). However, the mechanism of action of miR-140-3p in osteosarcoma remains unclear.

UBE2C is a member of the ubiquitin binding enzyme complex family, which promotes the degradation of a variety of abnormal or short-lived proteins during mid/late transformation of the cell cycle (13, 14). Overexpression of UBE2C can lead to typical tumor features in cells, including abnormal chromosome segregation, genomic instability, and changes in the cell cycle (15). Evidence suggests that UBE2C and the interaction between miRNA and UBE2C are closely related to the progression of various malignant tumors. Studies found that the UBE2C/CDH1/DEPTOR axis can regulate the cell cycle progression and autophagy of tumor cells. And confirmed that UBE2C may be an attractive new target for the treatment of lung cancer (16). Also, miR-525-5P realizes its inhibiting effect on the development of cervical cancer mainly by affecting the expression of UBE2C (17). However, the effect of miRNA interaction with UBE2C on the proliferation and metastasis ability of osteosarcoma cells remains to be further studied.

Therefore, we studied the role of miRNA-140-3p in osteosarcoma cells, and explored the mechanism by which miRNA-140-3p interacts with UBE2C to affect the malignant biological behavior of osteosarcoma cells. In conclusion, we hope that the conclusions of our study will provide new insights for improving the treatment of osteosarcoma.

## 2 Materials and methods

### 2.1 Data acquisition

Osteosarcoma related scRNA-seq data from GEO database (http://www.ncbi.nlm.nih.gov/geo/) and GARGET database (https://www.cancer.gov/ccg/research/genome-sequencing/target) were obtained and used for bioinformatics analysis. The R software package “DESeq 2” was used for differential analysis of dataset GSE28423, and the significance criterion was |logFC|> 2 and P value < 0.05 to screen for differentially expressed miRNAs. Pan-cancer survival analysis of miR-140-3p was performed using the UALCAN tool (http://ualcan.path.uab.edu/). With that, based on miRNA Target prediction database (miRDB-MicroRNA Target (https://mirdb.org/), microT-CDS V5.0 (https://dianalab.e-ce.uth.gr/microtv5/), miRPathDB (https://dianalab.e-ce.uth.gr/html/diana/web/index.php?r=mirpathv3), miRCarta (https://mircarta.cs.uni-saarland.de)) screened key downstream target genes. The R software package “DESeq 2” was used for differential analysis of datasets GSE28424 and GSE33382. The significance criterion was |log FC| > 2 and P value < 0.05 to screen for key mRNAs. Pan-cancer analysis of UBE2C was performed using the UALCAN tool (http://ualcan.path.uab.edu/).

### 2.2 Cell culture

Human osteoblast cells (hFOB 1.19), non-metastatic osteosarcoma cell lines (MG63, HOS) and highly metastatic cell lines (143B, K7M2) were purchased from Panocai Life Technology Co., LTD. (Wu Han, China). The hFOB 1.19 cells were cultured in DMEM/F-12 supplemented with 10% fetal bovine serum (FBS) (SERANA, Australia), 2.5 mM L-glutamine (Meeck, Germany), and 0.3 mg/mL genomycin (Gibco, USA), 34°C, 5% CO2 humidification incubator. Osteosarcoma cells were cultured in DMEM medium supplemented with 1% penicillin/streptomycin (Gibco, USA) and 10% FBS in a humidified incubator at 37°C and 5% CO_2_. The culture medium was replaced every 2 to 3 days. Cell passage was carried out when the cell fusion reached about 90%, and cells with good growth state and logarithmic growth stage were selected for the experiment.

### 2.3 Animal models

The animal experiment program was approved by the Ethics Committee of Experimental Animal Center of China Medical University (approval number: 20231000). 4-week-old female BALB/c nude mice without thymus were purchased from Huafukang (Beijing, China) and raised in an FPS environment. Adaptive feeding was followed for one week. The subcutaneous xenotransplantation model was established by injecting 2×10^6^ 143B cells suspended in 100 µL sterile 1×PBS buffer into the underarm of the right limb of nude mice (n=3 mice per group). The tumor volume was measured every 3 days and calculated using the following formula: Volume=length x width^2^×0.5. To establish a lung metastasis model, 2×10^6^ treated 143B cells were suspended in 200 µL 1×PBS buffer, and the above cells were injected into nude mice (n=3 mice per group) through the tail vein to simulate the osteosarcoma lung metastasis model. To evaluate the role of miR-140-3p *in vivo*, based on the manufacturer’s recommendation (RiboBio, China), intratumoral injection of miR-140-3p agomir/antagomir and corresponding negative controls every 3 days (for subcutaneous xenograft tumor models) or intravenously via the tail vein (for pulmonary metastasis models) for 2 weeks. After four weeks, the mice were killed, subcutaneous tumor tissues and lung tissues were extracted, weighed and photographed for analysis, stored in 4% paraformaldehyde, and part of the lung tissues were used for HE staining. Lung metastases were counted from H&E-stained sections using ImageJ software. At least 5 sections per mouse were analyzed. According to animal euthanasia guidelines, all mice were euthanized by CO_2_ asphyxiation.

### 2.4 Supporting information

Further details of methods and materials are displayed in document ‘Supporting-Information’.

## 3 Results

### 3.1 miR-140-3p is low expressed in osteosarcoma cells and affects the prognosis

**Figure 1:**
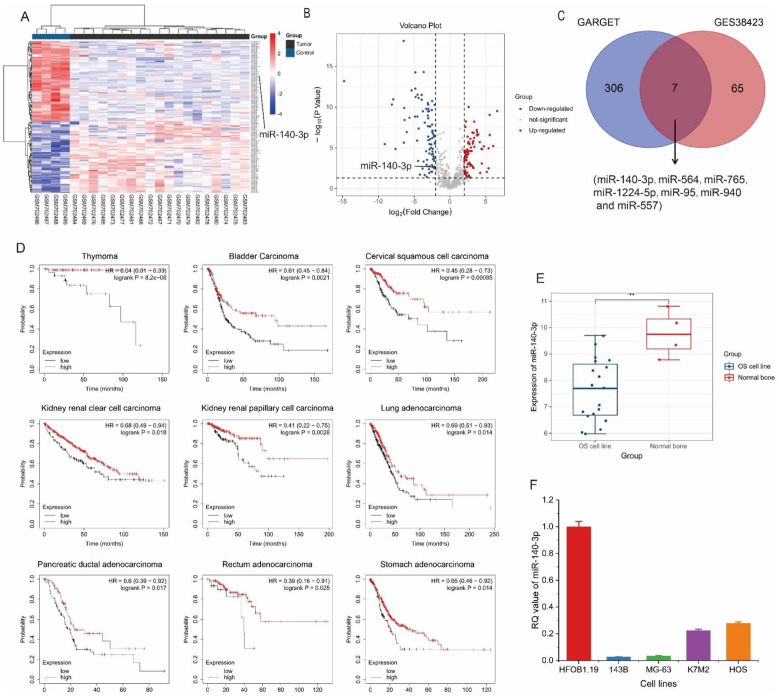
MiR-140-3p is low expression in osteosarcoma cells and is associated with the prognosis of tumor patients. **(A)** The cluster heat map showed 72 over-expressed miRNAs and 80 under-expressed miRNAs in the GSE28423 dataset of the GEO database (screening criteria: |logFC|< 2 and P value< 0.05). Rows show miRNAs, while columns show samples. Red and green bands represent up-regulated and down-regulated miRNAs, respectively. **(B)** Volcano maps showed 72 over-expressed miRNAs and 80 under-expressed miRNAs in the GSE28423 dataset of the GEO database (screening criteria: |logFC|< 2 and P value < 0.05). The red and green dots indicate up-regulated and down-regulated miRNAs, respectively. **(C)** Schematic diagram of overlapping target miRNAs with low differential expression in the SCNA-SEQ data from the GSE28423 dataset and the GARGET database. **(D)** Pan-cancer survival analysis of miR-140-3p was performed using UALCAN tool. **(E)** The expression of miR-140-3p in the GSE38423 dataset of GEO database was significantly lower in 19 osteosarcoma cell lines than in 4 normal bone tissues. **(F)** The expression of miR-140-3p in osteosarcoma cell lines (MG-63, 143B, HOS, K7M2) and normal osteoblasts (HFOB1.19) was determined by qRT-PCR. n.s., not significant; *P < 0.05; **P < 0.01; ***P < 0.001.

In order to explore the differentially expressed miRNAs in osteosarcoma cells, the scRNA-seq data in GEO database GSE28423 were analyzed using bioinformatics technology. A total of 152 differentially expressed miRNAs were found in osteosarcoma cells (screening criteria: |logFC|>= 2, and P value < 0.05), among which 72 miRNAs were overexpressed and 80 miRNAs were underexpressed (**Figures 1A, B**). In addition, we also analyzed the miRNA data of osteosarcoma patients in the GARGET database, and found that 313 miRNAs were overexpressed and 313 miRNAs were underexpressed in osteosarcoma tissues compared with normal bone tissues. The Venn diagram was used to analyze the results of the two databases, and the results showed that the expression of five miRNAs was up-regulated (miR-362-3p, miR-149, miR-96, miR-744, and miR-769-5p). The expression of seven miRNAs was down-regulated (miR-140-3p, miR-564, miR-765, miR-1224-5p, miR-95, miR-940, and miR-557) (**Figure 1C**). Pan-cancer analysis of the seven miRNAs with down-regulated expression showed that miR-140-3p was low in most cancers, and only high in KIRC and LIHC.

It is suggested that miR-140-3p may be a valuable biomarker for tumor prognosis (**Figure 1D**). Meanwhile, the expression of miR-140-3p in the GSE38423 dataset was significantly lower in osteosarcoma cells than in normal bone tissue (**Figure 1E**). The expression of miR-140-3p in osteosarcoma cell lines (MG-63, 143B, HOS, K7M2) and normal osteoblasts (HFOB1.19) was determined by qRT-PCR, and it was found that miR-140-3p was significantly underexpressed in osteosarcoma cells (**Figure 1F**). These evidences are sufficient to demonstrate that miR-140-3p is a key regulator in osteosarcoma cells.

### 3.2 MiR-140-3p mimics inhibits proliferation and metastasis of osteosarcoma cells

**Figure 2:**
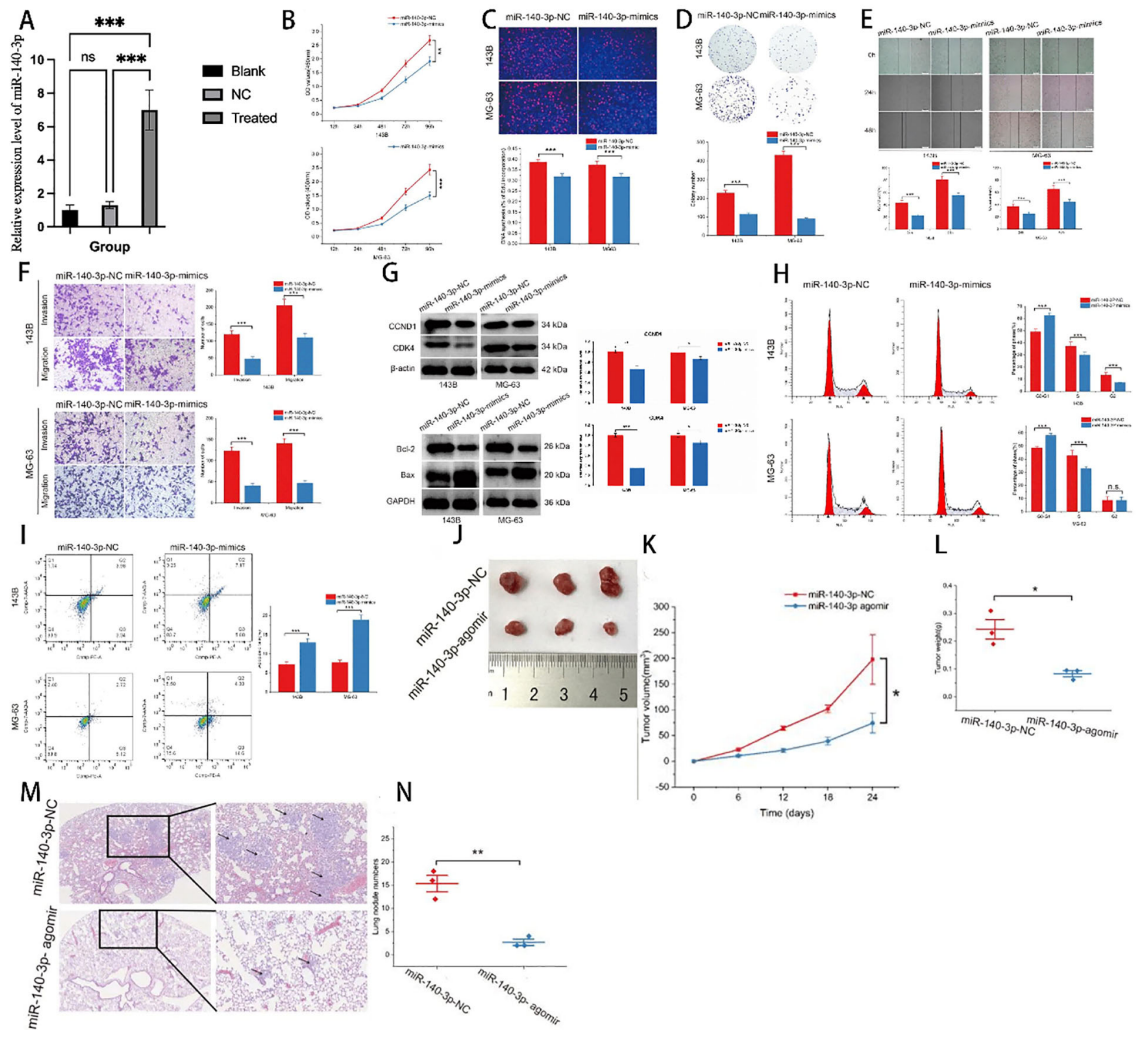
MiR-140-3p mimics inhibits proliferation and metastasis of osteosarcoma cells. **(A)** The qRT-PCR results of the miR-140-3p-overexpression versus untransfected cells in 143B cells. Similar results were obtained in MG-63 cells. **(B)** The effect of miR-140-3p mimics on the proliferation of 143B and MG-63 osteosarcoma cells was detected by CCK-8 assay. **(C)** EdU assay to detect the effect of miR-140-3p mimics on DNA synthesis in 143B and MG-63 osteosarcoma cells (scale, 100 µm). **(D)** The effect of miR-140-3p mimics on colony-forming ability of 143B and MG-63 osteosarcoma cells was detected by clonal formation assay. **(E)** The effect of miR-140-3p mimics on wound healing ability of 143B and MG-63 osteosarcoma cells detected by scratch assay (scale, 100 µm). **(F)** The effects of miR-140-3p mimics on the migration and invasion ability of 143B and MG-63 osteosarcoma cells were detected by Transwell migration and invasion assay (scale, 100 µm). **(G)** Western blotting was performed to evaluate the effects of miR-140-3p mimics on the expression of cell cycle and apoptosis-related markers in 143B and MG-36 osteosarcoma. **(H)** The effect of miR-140-3p mimics on cell cycle distribution in osteosarcoma 143B and MG-36 was detected by flow cytometry. **(I)** The effect of miR-140-3p mimics on apoptosis of 143B and MG-36 osteosarcoma cells was detected by flow cytometry. **(J)** Representative image of the effect of subcutaneous injection of miR-140-3p agomir on subcutaneous allografted osteosarcoma in nude mice. K&L. Effects of miR-140-3p agomir on the growth volume and weight of subcutaneous osteosarcoma in nude mice. **(M)** Representative image of **(K, L)** staining of lung tissue sections in a nude mouse osteosarcoma lung metastasis model (scale, 1000 µm). **(N)** Effect of miR-140-3p agomir on the number of lung metastases of osteosarcoma in nude mice. n.s., not significant; *P < 0.05; **P < 0.01; ***P < 0.001.

In order to explore the role of overexpressing miR-140-3p in osteosarcoma, we transfected 143B and MG-63 osteosarcoma cells with miR-140-3p mimics. qRT-PCR verified the transfection efficiency of miR-140-3p in both 143B and MG-63 cells (representative data from 143B cells shown in **Figure 2A**; similar results were observed in MG-63 cells). The results of the CCK-8 experiment, EdU experiment and clonal formation experiment showed that miR-140-3p mimics significantly inhibited the proliferation ability (**Figure 2B**), DNA synthesis ability (**Figure 2C**) and colony formation ability (**Figure 2D**) of 143B and MG-63 cells compared with Mir-140-3P-NC cells. In addition, the results of the cell scratch assay, Transwell migration assay, and Transwell invasion assay showed that miR-140-3p mimics significantly decreased the scratch healing ability (**Figure 2E**), migration ability (**Figure 2F**), and invasion ability (**Figure 2F**) of 143B and MG-63 osteosarcoma cells. Western blot analysis showed that miR-140-3p mimics decreased the expression of Bcl-2, CCND1, and CDK4, while promoting Bax expression, although the effect on CCND1 and CDK4 in MG-63 cells was milder than in 143B cells (**Figure 2G**).

The results suggest that miR-140-3p mimics affect the cell cycle progression and apoptosis of 143B and MG-63 osteosarcoma cells due to the physiological effects of these proteins. Flow cytometry analysis also reveals that miR-140-3p mimics induce G0/G1 phase arrest (**Figure 2H**) and enhance apoptosis of 143B and MG-63 osteosarcoma (**Figure 2I**). *In vivo* experiments, subcutaneous tumor volume (**Figures 2J, K**) and tumor weight (**Figure 2L**) and the number of lung metastases (**Figures 2M, N**) in nude mice injected with miR-140-3p agomir were significantly reduced compared with controls. Overall, all these findings suggest that overexpression of miR-140-3p inhibits the proliferation and metastasis ability of osteosarcoma cells.

### 3.3 MiR-140-3p inhibitor promotes proliferation and metastasis of osteosarcoma cells

**Figure 3:**
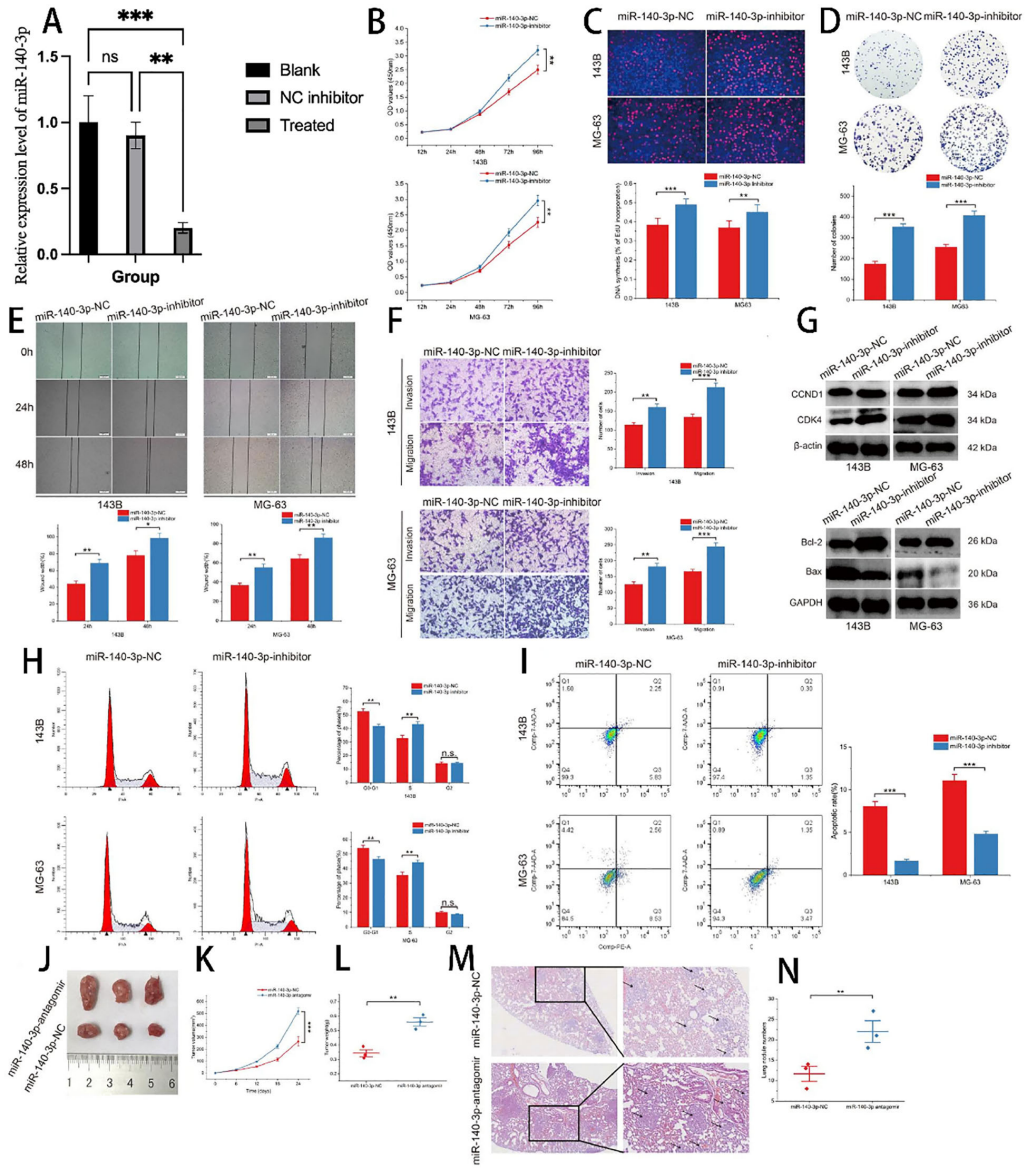
MiR-140-3p inhibitor promotes proliferation and metastasis of osteosarcoma cells. **(A)** The qRT-PCR results of the miR-140-3p inhibition in 143B cells. Similar results were obtained in MG-63 cells. **(B)** The effect of miR-140-3p inhibitor on the proliferation of osteosarcoma cells of 143B and MG-63 was detected by CCK-8 assay. **(C)** EdU assay detected the effect of miR-140-3p inhibitor on DNA synthesis capacity of 143B and MG-63 osteosarcoma cells (scale, 100 µm). **(D)** The effect of miR-140-3p inhibitor on colony formation ability of osteosarcoma cells 143B and MG-63 was detected by clonal formation assay. **(E)** The effect of miR-140-3p inhibitor on the wound healing ability of 143B and MG-63 osteosarcoma cells was detected by scratch assay (scale, 100 µm). **(F)** Transwell migration and Transwell invasion assay to detect the effect of miR-140-3p inhibitor on the migration and invasion ability of 143B and MG-63 osteosarcoma cells (scale, 100 µm). **(G)** Western blot was performed to evaluate the effect of miR-140-3p inhibitor on the expression of cell cycle and apoptosis-related markers of 143B and MG-63 osteosarcoma cells. **(H)** The effect of miR-140-3p inhibitor on cell cycle distribution of osteosarcoma 143B and MG-63 was detected by flow cytometry. **(I)** The effect of miR-140-3p inhibitor on apoptosis of 143B and MG-36 osteosarcoma cells was detected by flow cytometry. **(J)** Representative image of the effect of subcutaneous injection of miR-140-3p antagomir on subcutaneous allografted osteosarcoma in nude mice. **(K, L)** Effects of miR-140-3p antagomir on the growth volume and weight of subcutaneous osteosarcoma in nude mice. **(M)** Representative image of H&E staining of lung tissue sections in a nude mouse osteosarcoma lung metastasis model (scale, 1000 µm). **(N)** Effect of miR-140-3p antagomir on the number of lung metastases of osteosarcoma in nude mice. n.s., not significant; *P < 0.05; **P < 0.01; ***P < 0.001.

In order to explore the role of low expression of miR-140-3p in osteosarcoma, we transfected 143B and MG-63 cells with miR-140-3p inhibitor, and verified the transfection efficiency of miR-140-3p inhibitor by qRT-PCR. Osteosarcoma cell lines with low expression of miR-140-3p were successfully constructed (representative data from 143B cells shown in **Figure 3A**; similar results were observed in MG-63 cells). The results of the CCK-8 experiment, EdU experiment and clonal formation experiment showed that compared with miR-140-3p NC group, miR-140-3p inhibitor significantly promoted the proliferation capacity of 143B and MG-63 osteosarcoma cells (**Figure 3B**), DNA synthesis capacity (**Figure 3C**) and colony formation capacity (**Figure 3D**). In addition, the results of the cell scratch assay, Transwell migration assay and Transwell invasion assay showed that miR-140-3p inhibitor significantly enhanced the scratch healing ability (**Figure 3E**), migration ability (**Figure 3F**) and invasion ability (**Figure 3F**) of osteosarcoma cells 143B and MG-63. Western blot assay showed that miR-140-3p inhibitor promoted the expression of Bcl-2, CCND1 and CDK4, but inhibited the expression of Bax (**Figure 3G**). In view of the physiological effects of these proteins, these results indicated that miR-140-3p inhibitor can affect the cycle progression and apoptosis of 143B and MG-63 osteosarcoma cells. Flow cytometry analysis further revealed that miR-140-3p inhibitor significantly promoted the cycle progression of osteosarcoma cells (**Figure 3H**) and inhibited their apoptosis (**Figure 3I**). *In vivo* experiments showed that nude mice injected with miR-140-3p antagomir had a significantly increased subcutaneous tumor volume (**Figures 3J, K**) and weight (**Figure 3L**), as well as a higher number of lung metastases (**Figures 3M, N**), compared with controls.

Taken together, all these findings suggest that inhibiting the expression of miR-140-3p can promote the proliferation and metastasis ability of osteosarcoma cells.

### 3.4 UBE2C is a direct target gene of miR-140-3p and is associated with the prognosis of tumor patients

**Figure 4:**
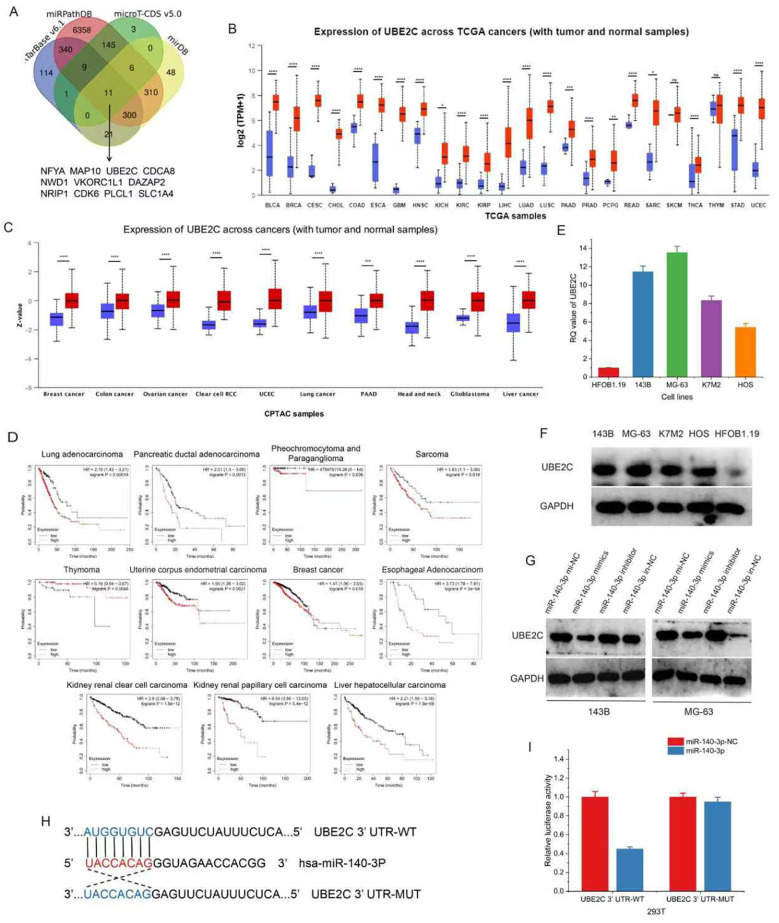
UBE2C is a direct target gene of miR-140-3p and is associated with the survival of tumor patients. **(A)** Four online databases: mirDB, microT-CDS v5.0, miRPathDB, and RTarBase v6.1 predict potential target gene outcomes of miR-140-3p. **(B, C)** Pan-cancer analysis of UBE2C expression. Asterisks indicate significant differences compared to corresponding normal tissues (Wilcoxon test, *P < 0.05, **P < 0.01, **P < 0.001). **(D)** Pan-cancer survival analysis of UBE2C was performed using the UALCAN tool. **(E)** The expression of UBE2C in osteosarcoma cell lines (MG-63, 143B, HOS, K7M2) and normal osteoblasts (HFOB1.19) was determined by qRT-PCR. **(F)** Western blotting was used to detect UBE2C expression in osteosarcoma cell lines (MG-63, 143B, HOS, K7M2) and normal osteoblasts (HFOB1.19). **(G)** The effects of miR-140-3p mimics and miR-140-3p inhibitor on UBE2C expression in 143B and MG-63 osteosarcoma cells were analyzed by Western blot assay. **(H)** Specific binding site between miR-140-3p and UBE2C. **(I)** Luciferase activity was evaluated in 293T cells after co-transfection with UBE2C-WT or UBE2C-MUT and miR-140-3p mimics or NC mimics.

In order to find the potential mechanism of action of miR-140-3p, four online databases, mirDB, microT-CDS v5.0, miRPathDB, and RTarBase v6.1 were used to predict the target genes of miR-140-3p, and 11 intersecting target genes were found in the prediction results of the four databases (**Figure 4A**). We performed bioinformatics analysis on the data in the GEO database GSE28423 and GSE33382 (screening criteria: |logFC|>2 and P value<0.05), it was found that UBE2C and CDC20 were the target genes shared with the target genes predicted by miR-140-3p in the online database. In addition, pan-cancer analysis of UBE2C was conducted using the UALCAN tool, and it was found that UBE2C was highly expressed in most cancer tissues (**Figures 4B, C**), and highly expressed UBE2C was negatively correlated with the survival rate of most tumor patients (**Figure 4D**). In osteosarcoma cells, mRNA (**Figure 4E**) and protein (**Figure 4F**) of UBE2C were highly expressed in osteosarcoma cells compared to normal bone cells. Next, by knockout or overexpression of miR-140-3p in 143B and MG-63 cells, it was found that the expression of UBE2C protein was significantly reduced after overexpression of miR-140-3p (**Figure 4G**). On the contrary, the expression of UBE2C protein was significantly increased after the deletion of miR-140-3p (**Figure 4G**). Potential binding sites for miR-140-3p and UBE2C were identified by predictive analysis of the TargetScan database (**Figure 4H**). These results suggest that UBE2C may be the target gene of miR-140-3p.

The results of dual luciferase gene reporting experiments showed that co-transfection of UBE2C-WT 3’-UTR fluorescent plasmid with miR-140-3p significantly changed fluorescence intensity (**Figure 4I**). Co-transfection of UBE2C-Mut 3’-UTR fluorescent plasmid with miR-140-3p did not change fluorescence intensity (**Figure 4I**). It was further confirmed that UBE2C is the direct target gene of miR-140-3p.

### 3.5 Overexpression of UBE2C can reverse the tumor inhibitory effect of miR-140-3p mimics

**Figure 5:**
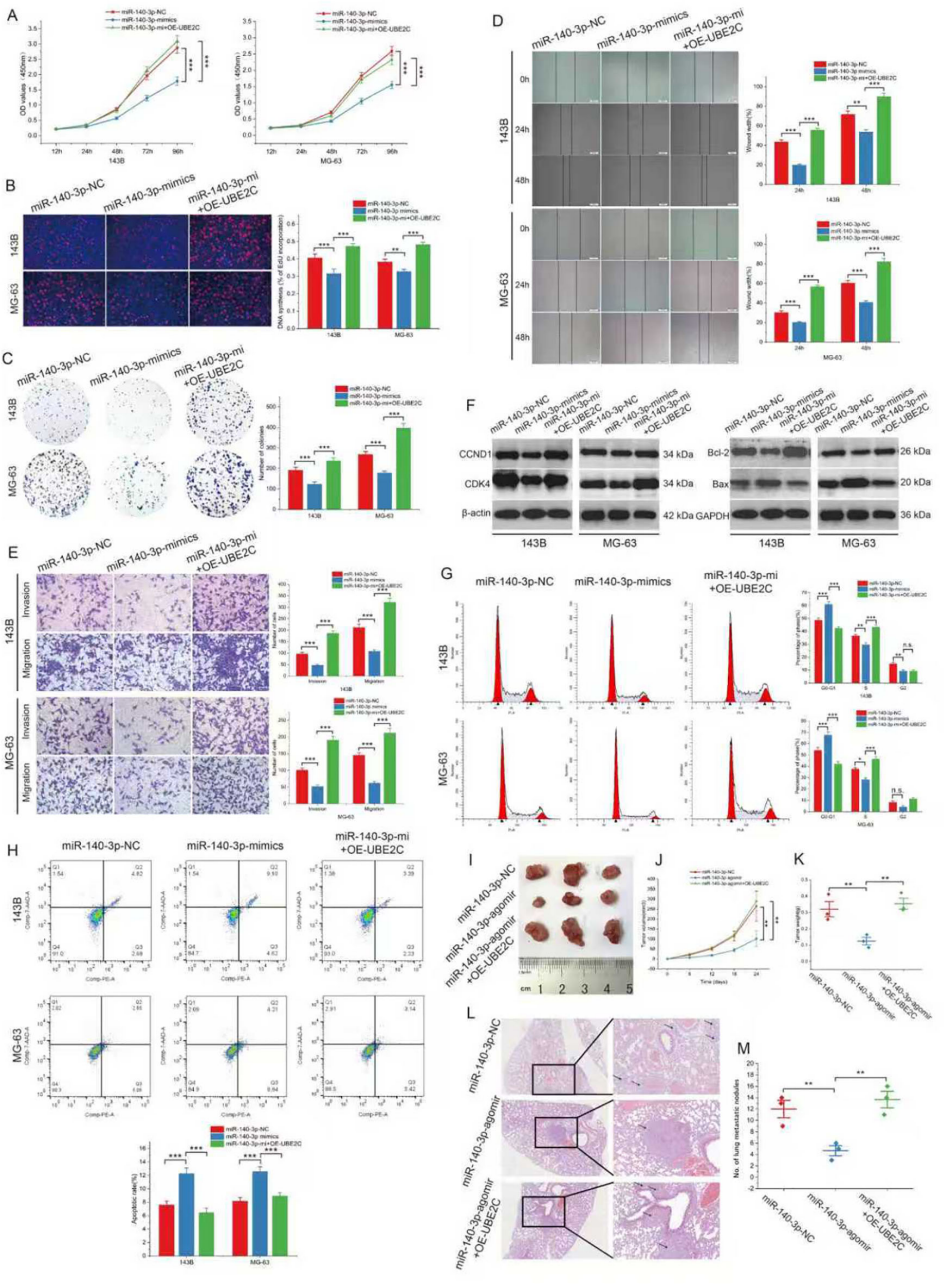
Overexpression of UBE2C can reverse the tumor inhibitory effect of miR-140-3p mimics. Osteosarcoma cells 143B and MG-63 were transfected with miR-140-3p-NC, miR-140-3p-mimics, and miR-140-3p-mimics and OE-UBE2C. **(A)** CCK-8 assay was used to evaluate the effect of UBE2C overexpression on the proliferation of miR-140-3p MIMics-mediated osteosarcoma cells. **(B)** EdU assay was performed to evaluate the effect of UBE2C overexpression on miR-140-3p MIMics-mediated DNA synthesis in osteosarcoma cells (scale, 100 µm). **(C)** The effect of UBE2C overexpression on the colony-forming ability of miR-140-3p MIMics-mediated osteosarcoma cells was evaluated by clonal formation assay. **(D)** Scratch assay was performed to evaluate the effect of UBE2C overexpression on miR-140-3p MIMics-mediated wound healing in osteosarcoma cells (scale, 100 µm). **(E)** Transwell migration assay and Transwell invasion assay were used to evaluate the effects of UBE2C overexpression on the migration and invasion ability of miR-140-3p MIMics-mediated osteosarcoma cells (scale 100 µm). **(F)** The effect of UBE2C overexpression on the changes of CCND1, CDK4, Bax and BCL-2 protein levels in osteosarcoma cells mediated by miR-140-3p mimics was analyzed by western blot assay. **(G)** The effect of UBE2C overexpression on cell cycle distribution mediated by miR-140-3p mimics in osteosarcoma was detected by flow cytometry. **(H)** The effect of UBE2C overexpression on miR-140-3p MIMics-mediated apoptosis of osteosarcoma cells was detected by flow cytometry. **(I)** Representative image of the effect of UBE2C overexpression on miR-140-3p Agomir-mediated osteosarcoma after subcutaneous transplantation in nude mice. **(J, K)** Effects of UBE2C overexpression on the growth volume and weight of miR-140-3p Agomir-mediated subcutaneous osteosarcoma in nude mice. **(L)** Representative images of lung tissue sections with H&E staining in osteosarcoma cell lung metastasis models in each group (scale, 1000 µm). **(M)** Weight of lung metastases of osteosarcoma cells in nude mice in each group. n.s., not significant; *P < 0.05; **P < 0.01; ***P < 0.001.

**Figure 6:**
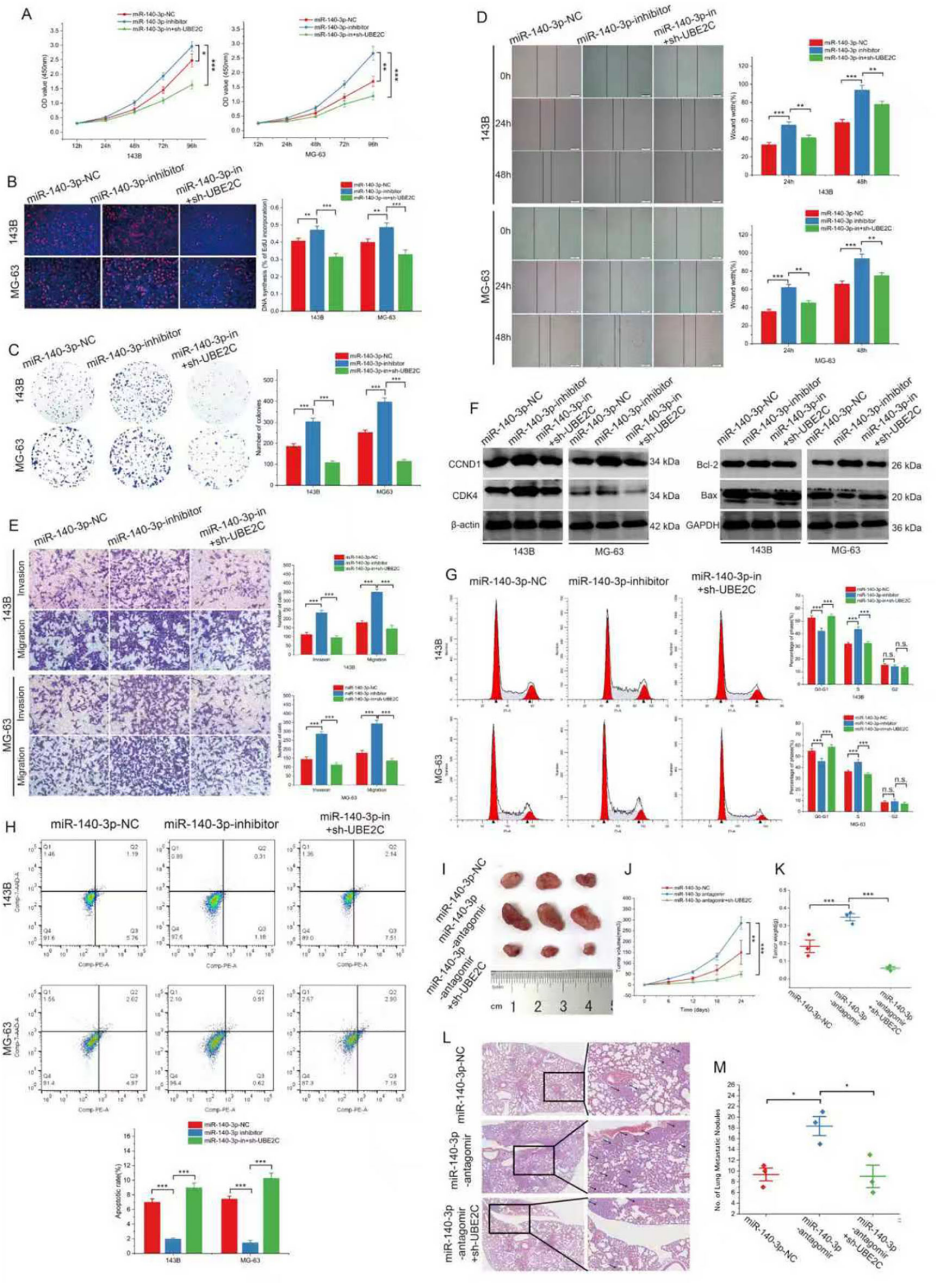
Low expression of UBE2C can reverse the cancer-promoting effect of miR-140-3p inhibitor. Osteosarcoma cells 143B and MG-63 were transfected with Mir-140-3P-NC, miR-140-3p inhibitor, miR-140-3p inhibitor and sh-UBE2C. **(A)** CCK-8 assay was used to evaluate the effect of low expression of UBE2C on the proliferation of osteosarcoma cells mediated by miR-140-3p inhibitor. **(B)** EdU assay was performed to evaluate the effect of low expression of UBE2C on DNA synthesis of osteosarcoma cells mediated by miR-140-3p inhibitor (scale, 100 µm). **(C)** The effect of low expression of UBE2C on colony formation ability of osteosarcoma cells mediated by miR-140-3p inhibitor was evaluated by clonal formation assay. **(D)** Scratch assay was performed to evaluate the effect of low UBE2C expression on miR-140-3p inhibitor-mediated cellular wound healing ability (scale, 100 µm). **(E)** Transwell migration assay and Transwell invasion assay were used to evaluate the effects of UBE2C low expression on the migration and invasion ability of osteosarcoma cells mediated by miR-140-3p inhibitor (scale, 100 µm). **(F)** The effect of low expression of UBE2C on the protein levels of CCND1, CDK4, Bax and BCL-2 of osteosarcoma cells mediated by miR-140-3p inhibitor was analyzed by western blot. **(G)** The effect of low expression of UBE2C on cell cycle distribution of osteosarcoma mediated by miR-140-3p inhibitor was detected by flow cytometry. **(H)** The effect of low expression of UBE2C on apoptosis of osteosarcoma cells mediated by miR-140-3p inhibitor was detected by flow cytometry. **(H)** Flow cytometry was used to detect the effect of UBE2C low expression on the apoptosis of osteosarcoma cells mediated by miR-140-3p inhibitor. **(I)** Representative image of the effect of low expression of UBE2C on miR-140-3p Agomir-mediated osteosarcoma after subcutaneous transplantation in nude mice. **(J, K)** Effects of low expression of UBE2C on the growth volume and weight of miR-140-3p Antagomir-mediated subcutaneous osteosarcoma in nude mice. **(L)** Representative images of lung tissue sections with H&E staining in osteosarcoma cell lung metastasis models in each group (scale, 1000 µm). **(M)** Number of lung metastases of osteosarcoma cells in nude mice in each group. n.s., not significant; *P < 0.05; **P < 0.01; ***P < 0.001.

In order to further verify that miR-140-3p can promote the proliferation and metastasis of osteosarcoma cells by targeting UBE2C, a series of experiments were conducted. Overexpression of UBE2C reverses these abilities of miR-140-3p mimics (**Figures 5A–C**). The results of the cell scratch test, Transwell migration test and Transwell invasion test showed that overexpression of UBE2C effectively weakened the inhibitory effect of miR-140-3p mimics on wound healing ability (**Figure 5D**), migration ability (**Figure 5E**) and invasion ability (**Figure 5E**) of osteosarcoma cells. Through CCK8 experiment (**Figure 6A**), EdU experiment (**Figure 6B**) and clonal formation experiment (**Figure 6C**), it was found that miR-140-3p mimics significantly reduced the proliferative ability, DNA synthesis ability and colony formation ability in 143B and MG-63 cells. In western blot experiments, miR-140-3p mimics down-regulated the expression of Bcl-2, CCND1, and CDK4 while increasing the expression of Bax, and overexpression of UBE2C restored the normal expression of these proteins (**Figure 6F**). Flow cytometry analysis showed that overexpression of UBE2C effectively impaired the ability of miR-140-3p mimics to significantly increase the proportion of cells in the G0/G1 phase and apoptosis (**Figures 6G,H**). *In vivo* experiments, overexpression of UBE2C reversed the inhibitory effects of miR-140-3p agomir on subcutaneous tumor volume growth (**Figures 6I, J**), tumor weight (**Figure 6K**), and lung metastasis capacity (**Figures 6L, M**) in nude mice. These findings suggest that overexpression of UBE2C can reverse the cancer-inhibiting effects of miR-140-3p mimics.

### 3.6 UBE2C knockdown can reverse the cancer-promoting effect of miR-140-3p inhibitor

To determine whether miR-140-3p promotes the proliferation and metastasis of osteosarcoma cells by targeting UBE2C, a series of experiments were performed. CCK8 experiment, EdU experiment and clonal formation experiment showed that miR-140-3p inhibitor significantly enhanced the proliferation, DNA synthesis and colony formation ability of 143B and MG-63 cells. However, UBE2C knockdown reversed these capabilities of miR-140-3p inhibitor (**Figures 6A–C**). The results of the cell scratch assay, Transwell migration assay and Transwell invasion assay showed that UBE2C knockdown also effectively weakened miR-140-3p inhibitor effect on wound healing capacity (**Figure 6D**), migration capacity (**Figure 6E**) and invasion capacity (**Figure 6E**) of osteosarcoma cells. In western blot experiments, miR-140-3p inhibitor increased the expression of Bcl-2, CCND1 and CDK4, while decreased the expression of Bax, and overexpression of UBE2C restored the normal expression of these proteins (**Figure 6F**). Flow cytometry analysis showed that UBE2C knockdown effectively impaired the ability of miR-140-3p inhibitor to significantly reduce the proportion of cells in the G0/G1 phase (**Figure 6G**) and apoptosis (**Figure 6H**). *In vivo* experiments, UBE2C knockdown could reverse the promoting effects of miR-140-3p antagomir on subcutaneous tumor growth (**Figures 6I, J**), tumor weight (**Figure 6K**), and lung metastasis capacity (**Figures 6L, M**) in nude mice. These findings suggest that UBE2C knockdown can reverse the cancer-promoting effects of miR-140-3p inhibitor.

### 3.7 MiR-140-3p affects the activation of the PI3K\Akt pathway by targeting UBE2C

**Figure 7:**
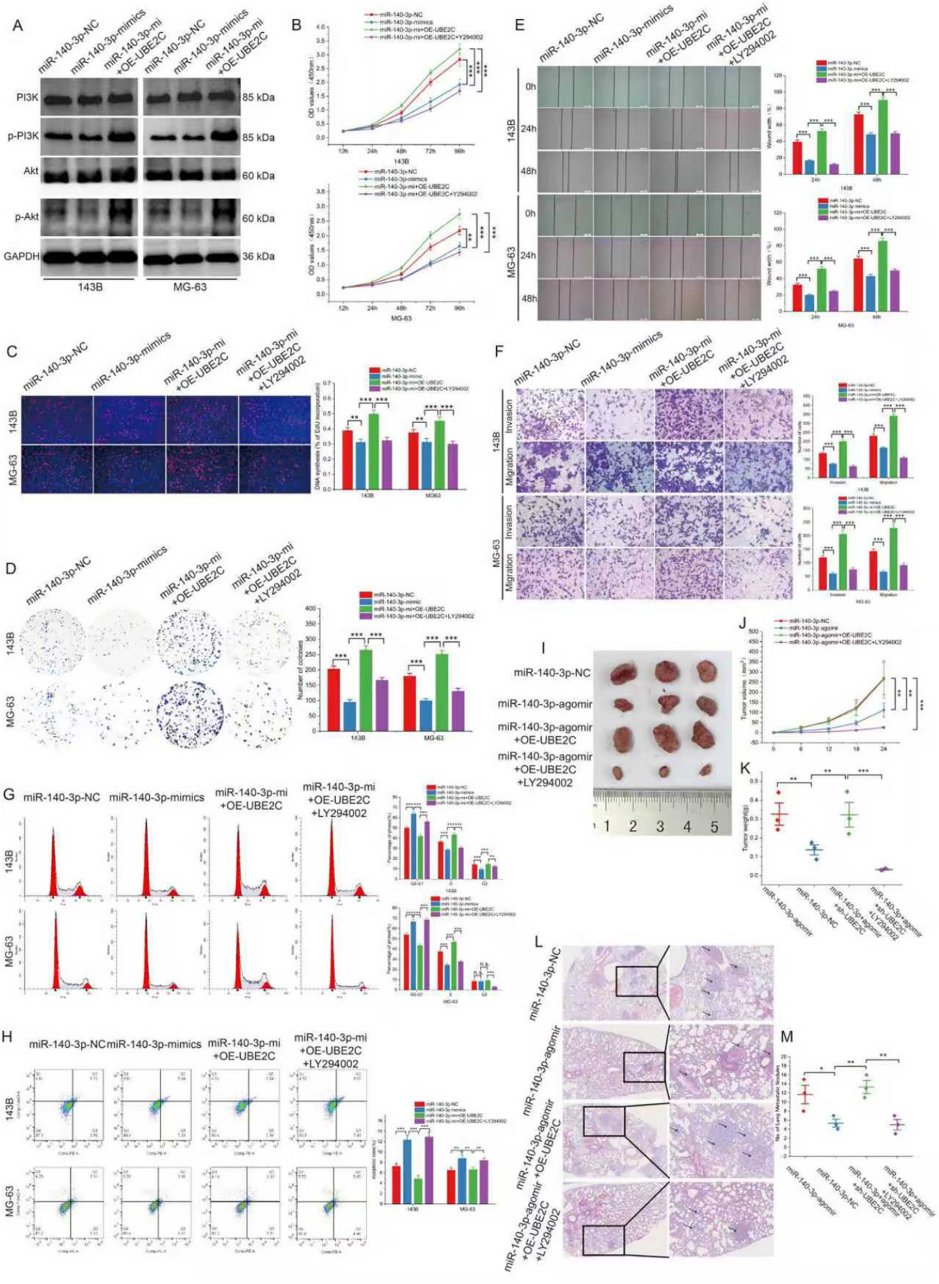
MiR-140-3p affects the activation of the PI3K-Akt pathway by targeting UBE2C and affects the proliferation and metastasis of osteosarcoma cells. Osteosarcoma cells 143B and MG-63 were transfected with miR-140-3p-NC, miR-140-3p-mimics, miR-140-3p-mimics+OE-UBE2C, miR-140-3p-mimics+OE-UBE2C+LY294002. **(A)** The effects of miR-140-3p mimics and the interaction between miR-140-3p mimics and UBE2C on the protein levels of p-PI3K, PI3K, p-Akt and Akt in osteosarcoma cells were analyzed by western blot assay, and normalized to the level of GAPDH. **(B)** CCK-8 assay was used to evaluate the effect of miR-140-3p/UBE2C/PI3K-Akt axis on the proliferation of osteosarcoma cells. **(C)** EdU assay was performed to evaluate the effect of miR-140-3p/UBE2C/PI3K-Akt axis on DNA synthesis capacity of osteosarcoma cells (scale, 100 µm). **(D)** The effect of miR-140-3p/UBE2C/PI3K-Akt axis on colony-forming ability of osteosarcoma cells was evaluated by clonal formation assay. **(E)** The effect of miR-140-3p/UBE2C/PI3K-Akt axis on the wound healing ability of osteosarcoma cells was evaluated by scratch assay (scale, 100 µm). **(F)** Transwell migration assay and Transwell invasion assay were used to evaluate the effects of miR-140-3p/UBE2C/PI3K-Akt axis on migration and invasion ability of osteosarcoma cells (scale, 100 µm). **(G)** The effect of miR-140-3p/UBE2C/PI3K-Akt axis on cell cycle distribution in osteosarcoma was determined by flow cytometry. **(H)** The effect of miR-140-3p/UBE2C/PI3K-Akt axis on apoptosis of osteosarcoma cells was detected by flow cytometry. **(H)** The effect of miR-140-3p/UBE2C/PI3K-Akt axis on apoptosis of osteosarcoma cells was determined by flow cytometry. **(I)** Representative images of osteosarcoma affected by subcutaneous transplantation of osteosarcoma cells in nude mice in each group. **(J, K)** Effect of miR-140-3p/UBE2C/PI3K-Akt axis on the growth volume and weight of subcutaneous tumor in osteosarcoma cells in nude mice. **(L)** Representative images of lung tissue sections with H&E staining in osteosarcoma cell lung metastasis models in each group (Scale, 1000 µm). **(M)** Number of lung metastases of osteosarcoma cells in nude mice in each group. n.s., not significant; *P < 0.05; **P < 0.01; ***P < 0.001.

As is widely known, an abnormal PI3K\Akt pathway is a common cause of tumorigenicity20. Recent studies have found that UBE2C can affect the activation of the PI3K\Akt pathway. Therefore, we speculate that miR-140-3p can affect the activation of the PI3K\Akt pathway through UBE2C in osteosarcoma, leading to changes in the proliferation and metastasis ability of osteosarcoma cells. To verify our hypothesis, we set up four groups of osteosarcoma cells: miR-140-3p MIMicS-NC, miR-140-3p mimics, miR-140-3p mi+OE-UBE2C, miR-140-3p mi+OE-UBE2C+LY294002. Western blot experiments showed that compared with the miR-140-3p MIMics-NC group, the ratio of p-PI3K\PI3K to pAkt\Akt in the miR-140-3p mimics group was significantly reduced. In the UBE2C overexpression group, the ratio of p-PI3K\PI3K to p-Akt\Akt could be restored to normal, but after the administration of P-inhibitor LY294002, the ratio of p-PI3K\PI3K to p-Akt\Akt was significantly reduced (**Figure 7A**). This suggests that the PI3K\Akt pathway can be affected by miR-140-3p and UBE2C. Through CCK8 assay, EdU assay, and clonal formation, it was found that the PI3K inhibitor LY294002 reversed the increase in the proliferation capacity of 143B and MG-63 cells (**Figure 7B**), DNA synthesis capacity (**Figure 7C**), and colony formation capacity (**Figure 7D**) caused by UBE2C overexpression. The results of the cell scratch assay, Transwell migration assay, and Transwell invasion assay showed that the PI3K inhibitor LY294002 effectively weakened the promoting effect of UBE2C on the wound healing ability (**Figure 7E**), migration (**Figure 7F**), and invasion ability (**Figure 7F**) of osteosarcoma cells. Flow cytometry analysis showed that miR-140-3p mimics induced an increase in the proportion of G0/G1 phase and apoptotic cells, and the overexpression of UBE2C could lead to the opposite result, while the PI3K inhibitor LY294002 could significantly increase the proportion of G0/G1 phase and apoptotic cells (**Figures 7G, H**). *In vivo* experiments, overexpression of UBE2C could reverse the inhibitory effects of miR-140-3p agomir on subcutaneous tumor growth (**Figures 6I, J**), tumor weight (**Figure 6K**) and lung metastasis capacity (**Figures 6L, M**) in nude mice. The PI3K inhibitor LY294002 reversed these effects caused by UBE2C overexpression (**Figures 6I–M**).

These findings suggest that miR-140-3p can affect the activation of the PI3K\Akt pathway through UBE2C, thereby affecting the proliferation and metastasis capacity of osteosarcoma.

## 4 Discussion

Osteosarcoma lacks specific early diagnostic biomarkers and therapeutic agents, resulting in unsatisfactory survival rates for patients. However, recent studies have found a close relationship between the development of osteosarcoma and abnormal miRNA expression. For example, miR-144-3p inhibits the progress of osteosarcoma cells through negative regulation of ZIB1 expression (18). The detection of serum miR-27A levels can be used as a biomarker in patients (19). However, the expression profiles and mechanisms of many miRNAs in osteosarcoma have not yet been fully understood.

In our study, bioinformatics analysis showed that the expression of miR-140-3p in bone tissue and osteosarcoma cell lines was lower than that in normal bone tissue, and low levels of miR-140-3p were associated with poor prognosis in tumor patients. We detected miR-140-3p in osteosarcoma cells, and found miR-140-3p was under-expressed in osteosarcoma cells. The above evidence suggests miR-140-3p plays an important role in the development of osteosarcoma. In order to further explore the role of miR-140-3p in osteosarcoma, miR-140-3p mimics were transfected into osteosarcoma cells, and it was found that over-expression of miR-140-3p significantly reduced the proliferation, DNA synthesis, wound healing, invasion and migration capabilities of osteosarcoma cells, and inhibited colony formation. This phenomenon was also discovered and confirmed in Qianchen Guo’s study, without further research (20). The over-expression of miR-140-3p in gastrointestinal tumors significantly reduced the expression of Bcl-2 in tumor cells and inhibited the malignant development of tumor cells (21, 22). In lung adenocarcinoma cells, up-regulation of miR-140-3p inhibits Bcl-2 and promotes Bax expression by targeting TYMS, leading to the proliferation, migration, invasion, and angiogenesis of lung adenocarcinoma cells (23). In our study, we found overexpression of miR-140-3p also reduced the expression of Bcl-2 and promoted the expression of Bax in osteosarcoma. Bax and Bcl-2 belong to the Bcl-2 gene family. Bax is a promotion gene of apoptosis, and Bcl-2 is a suppressor (24, 25). The results showed that overexpression of miR-140-3p induced cell cycle arrest in osteosarcoma cells. In addition, overexpression of miR-140-3p significantly decreased the expression of CCND1 and CDK4 in osteosarcoma cells. The expression changes of CCND1 and CDK4 are closely related to the cell cycle changes of osteosarcoma cells, and the combination of CCND1 and CDK4 is necessary for the G1/S transformation of the cell cycle (26, 27, 29, 30).

It is speculated that the overexpression of miR-140-3p can lead to the change of osteosarcoma cell cycle and enhance the apoptosis capacity of osteosarcoma cells. Flow cytometry further confirmed that overexpression of miR-140-3p induced G0/G1 phase stagnation and promoted apoptosis of osteosarcoma cells. However, the effect of miR-140-3p inhibitor in osteosarcoma cells was completely opposite to that of miR-140-3p mimics in osteosarcoma cells. The results of the *in vitro* experiment were also confirmed by subcutaneous tumor formation in nude mice and lung metastasis by tail vein injection. Thus, this study demonstrated for the first time that miR-140-3p acts as a tumor suppressor miRNA in osteosarcoma.

It has been proven that miRNAs regulate the expression of target genes by specifically binding to their target genes. Bioinformatics analysis of sequencing data from multiple databases revealed multiple potential target genes of miR-140-3p, including UBE2C, a member of the ubiquitin binding enzyme complex family. The UBE2C protein is a late promoting complex/ring (APC/C) -specific E2 ubiquitin binding enzyme that plays a key role by inactivating APC/C-dependent M-phase cell cycle progression (28, 31). UBE2C is overexpressed in many types of solid tumors, and the overexpression of UBE2C will accelerate tumor proliferation and metastasis. However, the regulatory mechanisms of UBE2C in osteosarcoma are not yet fully understood.

In this study, online database prediction and dual luciferase assay confirmed that UBE2C is negatively regulated by miR-140-3p, and UBE2C is expressed in various tumor tissues and osteosarcoma cell lines, and the expression of UBE2C is negatively correlated with miR-140-3p. Transfection of miR-140-3p mimics showed that miR-140-3p could inhibit the expression of UBE2C. In our study, a series of functional experiments further revealed that overexpression of UBE2C can reverse the inhibitory effects of miR-140-3p mimics on proliferation, DNA synthesis, colony formation, wound healing, invasion, and migration, as well as the promoting effects of G0/G1 phase arrest and apoptosis of osteosarcoma cells. UBE2C knockdown can reverse the promoting effects of miR-140-3p inhibitor on proliferation, DNA synthesis, colony formation, wound healing, invasion and migration, as well as G0/G1 phase block and apoptosis inhibition of osteosarcoma cells. In addition, we found that overexpression of miR-140-3p can significantly inhibit the activation of the PI3K/Akt pathway, while overexpression of UBE2C can promote the activation of the PI3K/Akt pathway. *In vitro* and *in vivo* experiments showed that the interaction between miR-140-3p and UBE2C affected the activation of the PI3K/Akt pathway. Moreover, the proliferation, DNA synthesis, colony formation, wound healing, invasion, migration, G0/G1 phase arrest and apoptosis of osteosarcoma cells were affected through the PI3K/Akt pathway. *In vivo* experiments also confirmed that the interaction between miR-140-3p and UBE2C affects the proliferation and metastasis of osteosarcoma cells *in vivo* by influencing the activation of the PI3K/Akt pathway. Although previous studies have implicated UBE2C in osteosarcoma tumor growth, our findings provide novel evidence that the miR-140-3p/UBE2C axis modulates the metastatic phenotype, particularly lung colonization, through the PI3K/Akt pathway. This represents a previously unrecognized mechanism linking miR-140-3p to metastatic progression in osteosarcoma.

Our study has several limitations. First of all, the subcutaneous tumor formation in nude mice and tail vein injection lung metastasis models used in this study have limitations, and the *in situ* tumor formation model is more convincing if used. Secondly, we only focused on the role of miR-140-3p in the proliferation and metastasis of osteosarcoma. Other factors related to tumor progression, diagnosis, and treatment, such as chemotherapy drug resistance, angiogenesis, and immune escape of tumors, have not yet been studied. Thirdly, the *in vivo* experiments were performed with three mice per group. Although *post-hoc* power analysis confirmed sufficient statistical power (>0.85) due to large effect sizes and low within-group variability, we recognize that larger cohorts would better capture biological variation. Therefore, our findings should be interpreted with caution, and future studies with expanded sample sizes are needed to further validate the conclusions.

**Figure 8:**
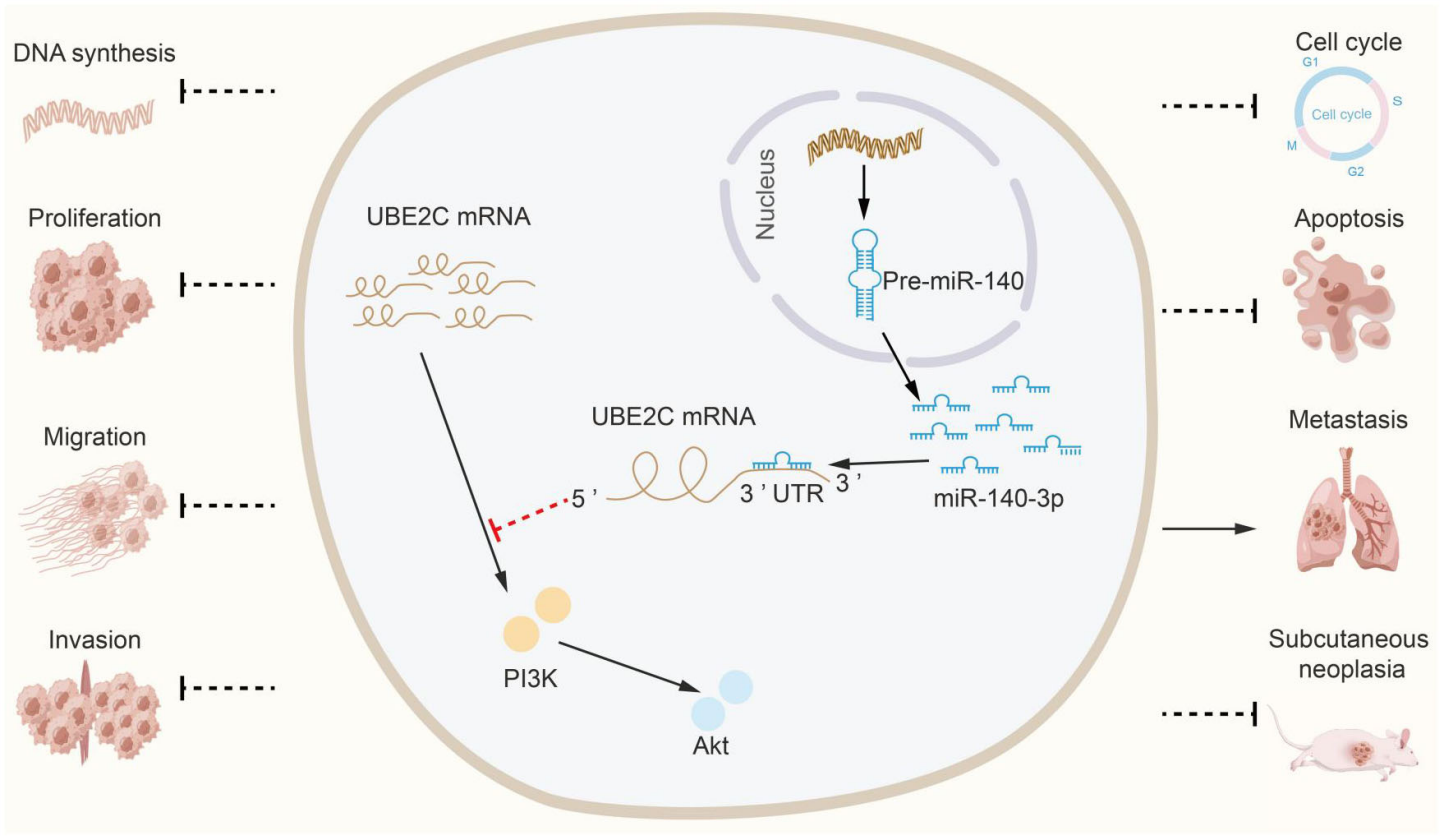
Schematic illustrating the biological role of the miR-140-3p/UBE2C/PI3K-Akt axis in osteosarcoma cells progression and metastasis.

In conclusion, this study suggests that the miR-140-3p/UBE2C/PI3K-Akt axis promotes the proliferation and metastasis of osteosarcoma cells. Our results demonstrate for the first time that miR-140-3p affects the PI3K/Akt pathway by targeting UBE2C in osteosarcoma cells, thereby contributing to the proliferation and metastasis of osteosarcoma cells, which may provide new insights for identifying potential biomarkers or therapeutic targets for osteosarcoma (**Figure 8**).

## Data Availability

The datasets presented in this study can be found in online repositories. The names of the repository/repositories and accession number(s) can be found in the article/supplementary material.
